# Low-Cost and Fast Epiretinal Membrane Detection and Quantification Based on SD-OCT

**DOI:** 10.1109/access.2025.3629332

**Published:** 2025-11-04

**Authors:** SEUNGJU BAEK, INSUP LEE, KUK JIN JANG, YONGSEOP HAN, JIN HYUN KIM

**Affiliations:** 1 Department of AI Convergence Engineering, Gyeongsang National University, Jinju 52828, South Korea; 2 Department of Computer and Information Science, University of Pennsylvania, Philadelphia, PA 19104, USA; 3 Department of Computer Engineering, Hongik University, Seoul 04066, South Korea; 4 Department of Ophthalmology, Changwon Hanmaeum Hospital, Changwon 51139, South Korea; 5 Department of AI Information Engineering, Gyeongsang National University, Jinju 52828, South Korea

**Keywords:** Medical AI, epiretinal membrane, YOLO, object detection, spectral domain OCT, disease quantification, retinal thickness

## Abstract

Epiretinal membrane (ERM) is a pathological condition characterized by the formation of a non-vascularized fibrocellular membrane on the inner retinal surface, leading to retinal traction, macular edema, and visual impairment. Optical coherence tomography (OCT) is the primary imaging modality for diagnosing macular ERM, enabling high-resolution visualization of retinal morphology and thickness. Although the transition from Time-Domain (TD-OCT) to Spectral-Domain OCT (SD-OCT) has significantly improved clinical imaging, the latest advancement—Swept-Source OCT (SS-OCT)—remains limited in clinical adoption due to its high cost, complexity, and processing demands. Consequently, many clinics are unable to leverage the intuitive en face visualization and quantitative analysis of ERM provided by SS-OCT. In this study, we propose a novel and cost-effective pipeline for ERM detection and quantification using only SD-OCT B-scans. Our method introduces a new en face projection technique, termed *Epiretinal Projection Image (EPI)*, which enables intuitive visualization of ERM spatial distribution. By leveraging a YOLOv11x-based deep learning model, we achieve high-precision ERM detection on B-scan images (mAP@50: 0.882, mAP@50:95: 0.556) and accurately project detected regions onto the EPI map for objective area quantification. Furthermore, we propose an association scoring mechanism that correlates EPI projections with retinal thickness maps, revealing a strong spatial relationship (correlation score: 0.771) between ERM presence and retinal thickening—a type of analysis not feasible even with SS-OCT. In the clinical reliability and expert acceptability evaluations conducted by board-certified retina specialists, the results demonstrated excellent inter-expert agreement (ICC = 0.94, *κ* = 0.89), with an average agreement score (AAS) of 4.77 and an acceptance rate (AR, scores ≥ 4) of 93.3%). These findings indicate a strong alignment between the model outputs and real-world clinical judgment. Our findings demonstrate that the proposed system provides accurate and interpretable ERM localization and quantitative assessment using widely available SD-OCT devices, suggesting its potential for reliable clinical application pending further large-scale validation.

## I. INTRODUCTION

Epiretinal membrane (ERM) is a condition in which an avascular fibroblastic membrane forms on the inner border of the retina, causing the fibroblastic membrane to contract and resulting in retinal folds or traction and impairing vision. The side-effect of various diseases, such as inflammatory diseases, retinal vascular disease, trauma, and retinal detachment, can cause ERM. However, most cases are idiopathic [1].

In many patients, the epiretinal membrane progressively worsens, resulting in symptoms such as decreased central vision, distorted vision, visual field disturbances, and inequality of vision. Some cases need surgical treatment, called vitrectomy, to remove the ERM.

Over the past 20 years, the prevalence of ERM in the global population has increased to 34.1%, with aging being a major risk factor [2], [3].

ERM is usually diagnosed with fundus photography and optical coherence tomography (OCT) [4]. OCT is a non-invasive test that identifies and analyzes the structures of the eye, including the retina and optic nerve papilla. Imaging the different retinal layers by OCT enables early detection and diagnosis of retinal diseases. OCT is highly accurate and widely used for detecting ERM at the inner retina.

Since its development in the 1990s, OCT has evolved rapidly [5], [6], improving resolution and speed [7], [8], [9]. Spectral-Domain OCT (SD-OCT) replaced Time-Domain OCT (TD-OCT) as the standard in clinical settings. SD-OCT offers faster acquisition and higher resolution, allowing for better visualization of fine retinal structures. It remains the most commonly used OCT device due to its lower cost and broad availability [10].

More recently, Swept-Source OCT (SS-OCT) has been developed. It uses longer wavelengths and faster scanning, offering deeper imaging and high-quality en face views, especially of the choroid [10], [11], [12], [13]. For ERM diagnosis, SS-OCT provides full en face visualization of the membrane and allows for better estimation of its area [14], [15], [16].

However, despite its advantages, SS-OCT adoption in clinics is still limited. Many hospitals and device manufacturers prefer SD-OCT because SS-OCT systems are expensive, require powerful processors, and increase data processing time [10], [17], [18]. SD-OCT, on the other hand, provides reliable B-scan images and is already integrated into most clinical workflows. As a result, SD-OCT remains the preferred tool in many settings.

Although B-scans in SD-OCT are not en face, they still contain rich structural information. If we can generate en face-like images from these B-scans, we could simulate SS-OCT results without needing expensive hardware. This would allow low-cost, fast, and accessible ERM visualization and quantification, bringing SS-OCT-level diagnostics to resource-limited settings.

In this study, we propose a new SD-OCT-based method that generates en face images and quantifies ERM area using deep learning and image processing. We introduce the *Epiretinal Projection Image (EPI)*, which combines the detection results from 50 SD-OCT B-scans to create a projection view of ERM distribution. This provides intuitive visualization and allows for accurate area measurement, similar to what SS-OCT offers.

Our model, based on YOLOv11x, achieves high detection accuracy (mAP@50: 0.882; mAP@50:95: 0.556), and can operate with low computational cost.

Moreover, we introduce a novel technique that quantifies the relationship between ERM location and retinal thickness, using an association score (correlation: 0.771). This feature is not available in current SS-OCT or SD-OCT systems. It helps reduce confusion in diagnosis, especially in cases with macular edema or thickness changes after surgery.

In summary, our work enhances the diagnostic power of SD-OCT, combining its affordability and speed with en face visualization and ERM-thickness quantification.

As shown in TABLE 1, our method leverages the strengths of both SD-OCT and SS-OCT, while avoiding their limitations. This provides a sustainable and scalable solution for ERM diagnosis and surgical planning, without the burden of upgrading to SS-OCT systems.

Our technology enhances the utility of the widely used SD-OCT and significantly reduces the time and diagnostic burden associated with ERM diagnosis for specialists. Additionally, our method of quantifying the relationship between ERM area and retinal thickness aids in accurately determining the status of ERM and guiding treatment decisions.

This paper is organized as follows: In Section II, we review related works focusing on the use of deep learning and SD-OCT for ERM detection and diagnosis, comparing previous methodologies and their outcomes. Section III outlines the proposed approach, detailing the process of generating en face images from SD-OCT B-scans, and highlights the improvements in ERM detection and quantification. In Section IV, we describe the use of YOLO models for ERM detection, where three different versions—YOLOv5, YOLOv8, and YOLOv11—were trained and evaluated. We also compare detection performance under different training dataset configurations to analyze the effect of data composition. Section V presents the creation of ERM projection images from SD-OCT B-scans and discusses how these projections can enhance diagnostic precision. In Section VI, we introduce our method for quantifying the ERM area using en face images and retinal thickness maps. Section VII provides an analysis of the association between ERM projection images and retinal thickness, discussing how the association influences treatment strategies. In Section IX, we address several limitations of the study and areas for future exploration. Finally, Section X concludes the paper by summarizing the findings and the potential impact of our approach on clinical practice.

## II. RELATED WORKS

There have been numerous previous studies that have applied deep learning techniques to the diagnosis of ERM using SD-OCT [19], [20]. They mainly used deep learning techniques to identify and localize ERMs in each SD-OCT B-scan image, and achieved a certain level of performance, demonstrating the feasibility of automatic diagnosis of ERM. These studies primarily utilized deep learning techniques to identify and localize ERM in individual SD-OCT B-scan images. They achieved notable performance levels, demonstrating the feasibility of automating the diagnosis of ERM. This approach showcases how machine learning can potentially streamline the diagnostic process, reduce human error, and improve the speed and accuracy of ERM detection. In [14], it is demonstrated that SS-OCT en face images are more intuitive and effective for diagnosing ERM compared to SD-OCT en face images. The entire area of the ERM, which was not clearly visible in SD-OCT en face images, could be distinctly identified in SS-OCT en face images, enhancing the visibility and accuracy of ERM detection. This finding underscores the advantages of SS-OCT technology in providing clearer and more detailed imaging for ERM diagnosis. Additionally, the [14] illustrated that by overlaying a retinal thickness map on a SS-OCT en face image, which displays the overall area of the ERM, it is possible to highlight areas where ERM traction increases retinal thickness. This technique enhances the visualization of the mechanical effects of ERM on the retina, aiding in a more comprehensive assessment and potentially guiding more targeted interventions. The [14] highlights the superiority of SS-OCT in diagnosing ERM, noting its advanced imaging capabilities. However, it also points out the limitations of SS-OCT, particularly its higher cost, which could restrict its widespread use in clinical practice due to budgetary constraints.

In this study, we aimed to harness the diagnostic advantages of SS-OCT for ERM detection by applying deep learning and image processing techniques to SD-OCT images. This approach seeks to improve the diagnostic capabilities of SD-OCT, making it more effective in identifying ERM, similar to the performance of SS-OCT.

## III. APPROACHES TO ERM DIAGNOSIS

### A. STUDY DESIGN

The protocol of this retrospective study was approved by the Institutional Review Board of Gyeongsang National University Hospital and the principles of the Declaration of Helsinki. The requirement for obtaining informed patient consent was waived by the institutional review board (GNUH 2022-01-032) due to the retrospective nature of the study.

### B. OUR APPROACH

Our approach to detecting and quantifying ERM begins with identifying ERM regions in SD-OCT B-scan images using a deep learning-based object detection model. Based on these detections, we generate an en face retinal image by projecting the detected ERM areas, thereby visualizing the regions affected by ERM across the entire retinal plane.

Subsequently, we quantify the total ERM area and evaluate its spatial correlation with the retinal thickness in the corresponding regions. This analysis aids ophthalmologists in making more informed and personalized decisions regarding ERM treatment.

**FIGURE 1.**
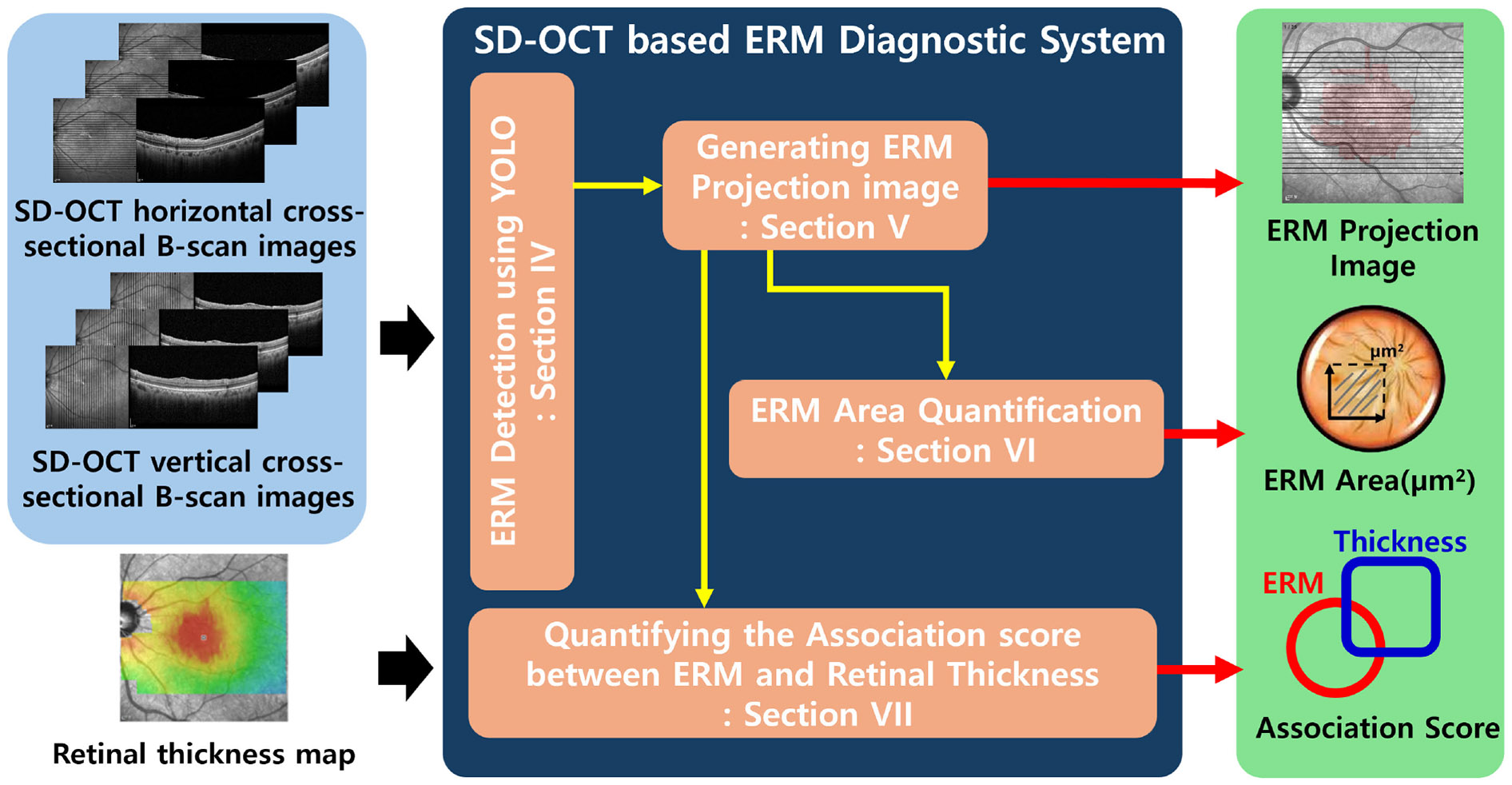
The overall architecture.

The overall architecture of the proposed pipeline is illustrated in FIGURE 1. The inputs consist of 50 SD-OCT B-scan images—25 horizontal and 25 vertical cross-sections—and a corresponding retinal thickness map that encompasses the same region.

First, we apply the YOLO object detection model to each of the 50 B-scan images to localize the ERM in every cross-sectional scan. We experimented with three different YOLO versions (YOLOv5, YOLOv8, and YOLOv11), and also analyzed the impact of training dataset composition on detection performance.

Second, the ERM regions detected across the B-scans are aggregated and projected onto a 2D en face plane to produce what we refer to as the *ERM Projection Image*. This image offers a comprehensive and intuitive visualization of the spatial extent of ERM.

Third, we quantify the area of ERM using the projection image and compare it against the corresponding regions in the retinal thickness map. This enables precise evaluation of the relationship between ERM and retinal swelling, which is essential for determining optimal treatment strategies.

Our approach allows for detailed assessment of the structural impact of ERM on the retina and provides a reliable tool for both visualization and quantitative analysis. By combining high-resolution SD-OCT data with deep learning and post-processing techniques, we offer a cost-effective and clinically valuable method for diagnosing and managing ERM.

### C. ERM DIAGNOSIS USING SD-OCT IMAGES

**FIGURE 2.**
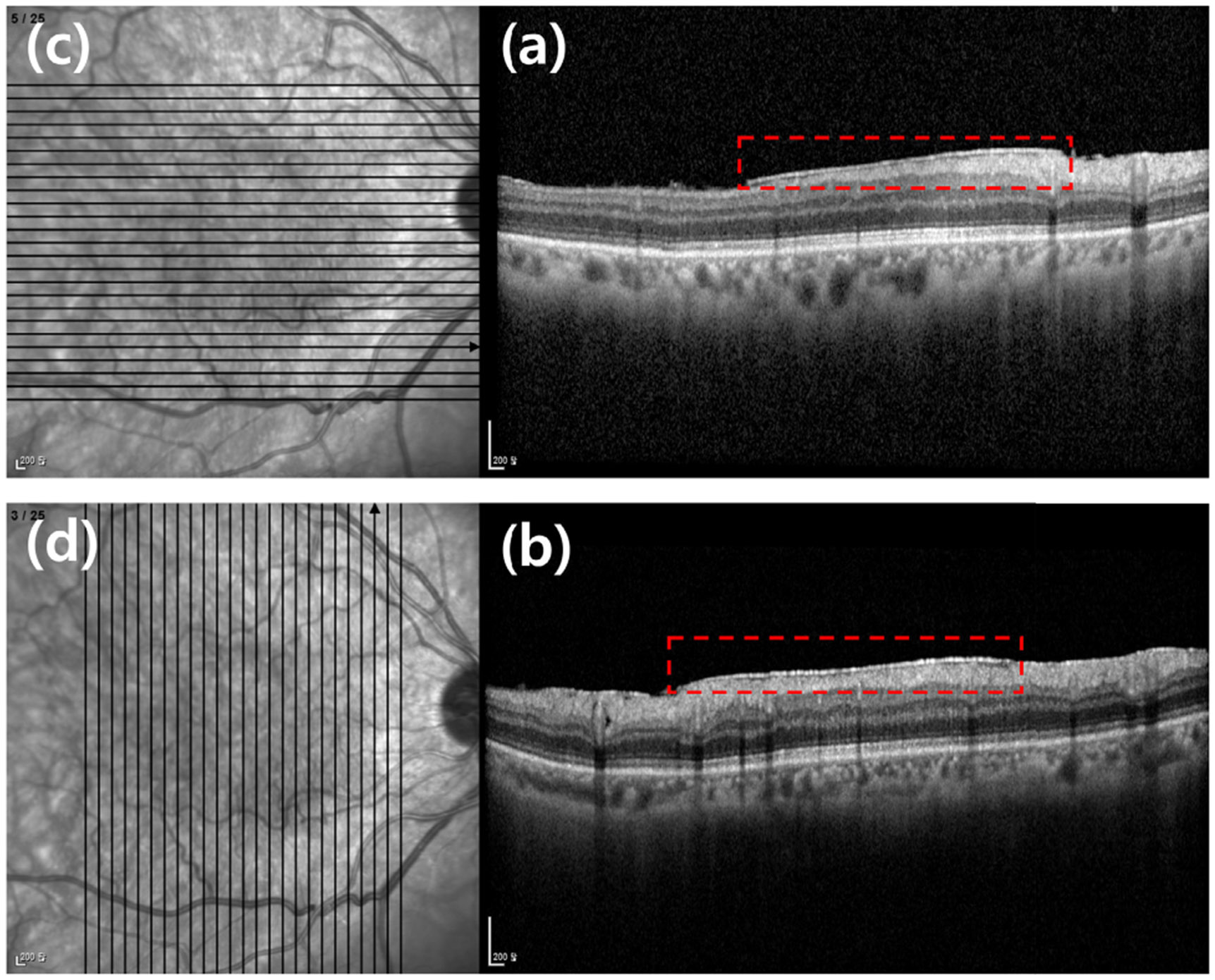
Two examples of ERM from SD-OCT are shown: (a) and (b) feature B-scan images, which are single 2D cross-sectional views that enable observation of the retinal layer structure at a specific foveal point in the eye. (c) and (d) show en face images (C-scan images), which provide a view from the front of the retina. This view is particularly useful for assessing the location, size, and distribution of lesions.

SD-OCT is the most popular method for determining the location and area of the epiretinal membrane. The SD-OCT device used in this study generates two types of images: B-scan images and en face images, also known as C-scan images. FIGURE 2 presents two examples of SD-OCT images, each consisting of a pair of images: an en face image on the left and a B-scan image on the right. Each en face image (c) and (d) shows most of the retina from the eye and indicates the orientation and location of the retinal cross-sections where the B-scan images (a) and (b) are taken, using a vertical or horizontal line.

In this study, we used 50 B-scan images from a single eye, consisting of 25 B-scan images of horizontal cross-sections and 25 B-scan images of vertical cross-sections. ERM can be diagnosed by identifying abnormal layers on the outermost part of the retina in cross-sectional B-scan images. It allows for precise detection and assessment of ERM. In FIGURE 2, the presence of epiretinal membrane is observed in images FIGURE 2 (a) and (b), as highlighted by the red bounding boxes. To diagnose ERM and develop a more effective treatment plan, the presence of ERM is individually assessed across 50 retinal cross-sections in the B-scan images. Compared to SD-OCT en face images, which may not provide clear diagnostic information in cases with multiple eye diseases or when ERM boundaries and areas are unclear, SD-OCT B-scan images offer higher disease identification accuracy due to their detailed cross-sectional views that clearly depict retinal structures and abnormalities. However, B-scan images have the drawback of being less intuitive compared to en face images, which provide an overall view of the macular surface, as they require examining 50 individual cross-sections of the macula. This process is also more time-consuming.

In diagnosing ERM, the retinal thickness map is effectively used together SD-OCT images. In the following section, we will discuss how these two images, with their different viewpoints, work together to diagnose ERM.

### D. ERM DIAGNOSIS USING RETINAL THICKNESS MAP

**FIGURE 3.**
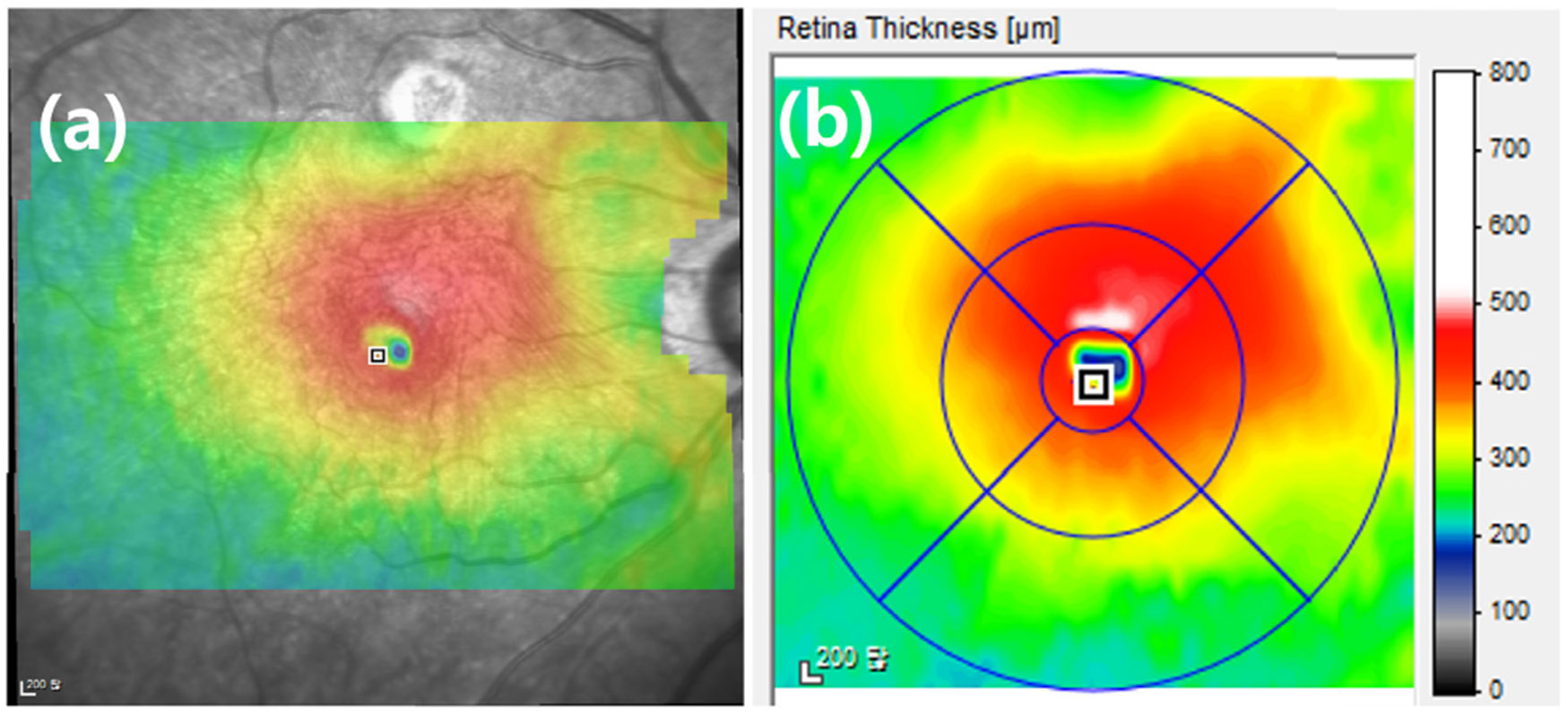
Retinal Thickness map example: (b) is a color visualization of the thickness of the retinal layers in *μ*m. The thickness of the retinal layer corresponding to the color can be found in the rightmost color bar. (a) shows the retinal thickness in the macular region overlaid on the en face image, allowing you to observe the retinal thickness and ocular tissues together.

ERM forms on the surface of the retina, and as it matures, it tends to contract or shrink. This shrinking of the ERM pulls on the underlying retinal layers, resulting in a gradual thickening of the retina in the affected area. Since thickened retinal layers can cause vision distortion and impairment, it is important to find the best treatment by correlating the presence of ERM with retinal thickness. A retinal thickness map is a color-coded image that shows the thickness of the retinal layers in the macula, offering a visual representation of thickness variations across different areas. The thickness map is often used together with B-scan images, which provide detailed structural information about the retinal layers (The thickness map is different from C-scan images which is not in colored-code image) This combination is especially valuable in diagnosing ERM, as it helps assess the area and distribution of retinal thickening, thereby improving diagnostic accuracy. FIGURE 3.(a) shows areas of the retina that appear thicker, likely indicating thickening of the retinal layers due to ERM.

However, in patients with conditions like macular edema, macular degeneration, or other macular diseases, as well as in cases where the retina remains swollen after ERM treatment, the retinal thickness map may show thickening of the retinal layers similar to what is observed with ERM. In clinical practice, the diagnosis of ERM is conducted alongside an analysis of retinal thickness maps from B-scan images to distinguish whether retinal thickness changes are due to ERM or other causes.

In short, ERM should be diagnosed by combining the thickness map with B-scan images. However, the thickness map provides an en-face view, while the B-scan image offers a cross-sectional view, meaning their perspectives are orthogonal. As a result, the eye surgeon must interpret these two separate images from different viewpoints to determine the best surgical or treatment approach. It consumes time and seems less intuitive. For this reason, this paper presents a way of transform the view point of B-scan images for ERM diagnosis to the same view point of the thickness map so that their view point is aligned to provide a more intuitive decision from the combined view point of the images.

## IV. ERM DETECTION USING YOLO MODEL

In this section, we present our approach to detecting ERM in B-scan images of SD-OCT data using a state-of-the-art YOLO-based object detection model. To identify the most suitable model for this task, we conducted comparative experiments using three different YOLO versions—YOLOv5, YOLOv8, and the recently released YOLOv11—under varying training dataset sizes (1,300 and 2,200 images), with a fixed evaluation set of 650 images.

### A. YOLOv5, YOLOv8, AND YOLOv11: MODEL COMPARISON FOR ERM DETECTION

We experimentally evaluated YOLOv5, YOLOv8, and YOLOv11 to determine the most effective model for detecting abnormal layers on the outer surface of the retina in SD-OCT B-scan images. These models are widely used for real-time object detection and are capable of predicting object locations and classes within a single output layer.

YOLOv8 and YOLOv11, the more recent versions, incorporate architectural enhancements such as feature map fusion, upsampling, and advanced backbone designs, which offer improvements in both accuracy and inference speed over YOLOv5. Despite their lightweight convolution-based architecture, all models performed well in complex OCT image environments.

### B. TRAINING AND EXPERIMENTATION SETTINGS

We trained and evaluated the models using a total of 2,850 SD-OCT B-scan images, annotated under the supervision of ophthalmologists at Changwon Gyeongsang National University Hospital. Two sets of training data (1,300 and 2,200 images) were used to evaluate the impact of training size, while a common evaluation set of 650 images was used across all experiments.

Images were resized to 640×640 resolution. Training was conducted using PyTorch on a Windows 10 environment with an Intel i7-11700K@3.60GHz CPU and an NVIDIA GeForce RTX 4080 16GB GPU, with CUDA 11.8 and Python 3.9.

For each YOLO version, we tested four pre-trained model variants (s, m, l, x) based on the COCO dataset, each with varying depths and complexities.

### C. EVALUATION OF YOLO MODELS

TABLE 2 summarizes the best evaluation results of each YOLO model on ERM detection, measured at the optimal epoch. It also includes the model size in parameters and the effect of training dataset size (Full: 2,200 images, Half: 1,300 images). “Train Dataset Size” refers to the number of B-scan images used for training, and the model performance is evaluated on a separate fixed test set.

Precision measures how many predicted positives are actually correct, while recall measures how many of the true positives are correctly predicted. Although these metrics do not scale proportionally with model size, the combined metric mAP—especially mAP@50:95—tends to increase with model complexity. This suggests that larger models are better suited to rigorous evaluation conditions with higher Intersection over Union (IoU) thresholds.

Among all model versions, YOLOv11x demonstrated the highest overall detection performance, achieving the best values in both mAP@50 and mAP@50:95. While it involves a larger model size compared to earlier versions, its detection accuracy and robustness make it particularly well-suited for identifying ERM in SD-OCT B-scan images. Despite the increased model complexity, the inference time remains significantly faster than manual diagnosis by ophthalmologists and can be optimized further depending on the deployment environment.

These results reveal several key insights. First, detection performance generally increases with model complexity, especially in terms of mAP@50:95, highlighting the advantage of deeper and more expressive architectures such as YOLOv11x. Second, increasing the training dataset size from 1,300 to 2,200 images consistently improves performance across all models, underscoring the critical role of data sufficiency in training robust detectors. Third, while smaller variants (e.g., YOLOv5s, YOLOv8s) yield lower mAP scores, they offer potential for deployment in low-resource environments due to their lightweight nature. Finally, in medical image analysis, mAP@50:95 is particularly important as it reflects precise localization—a crucial factor in clinical diagnosis. These observations collectively support the use of YOLOv11x with full dataset training as the optimal configuration for ERM detection in SD-OCT images.

**FIGURE 4.**
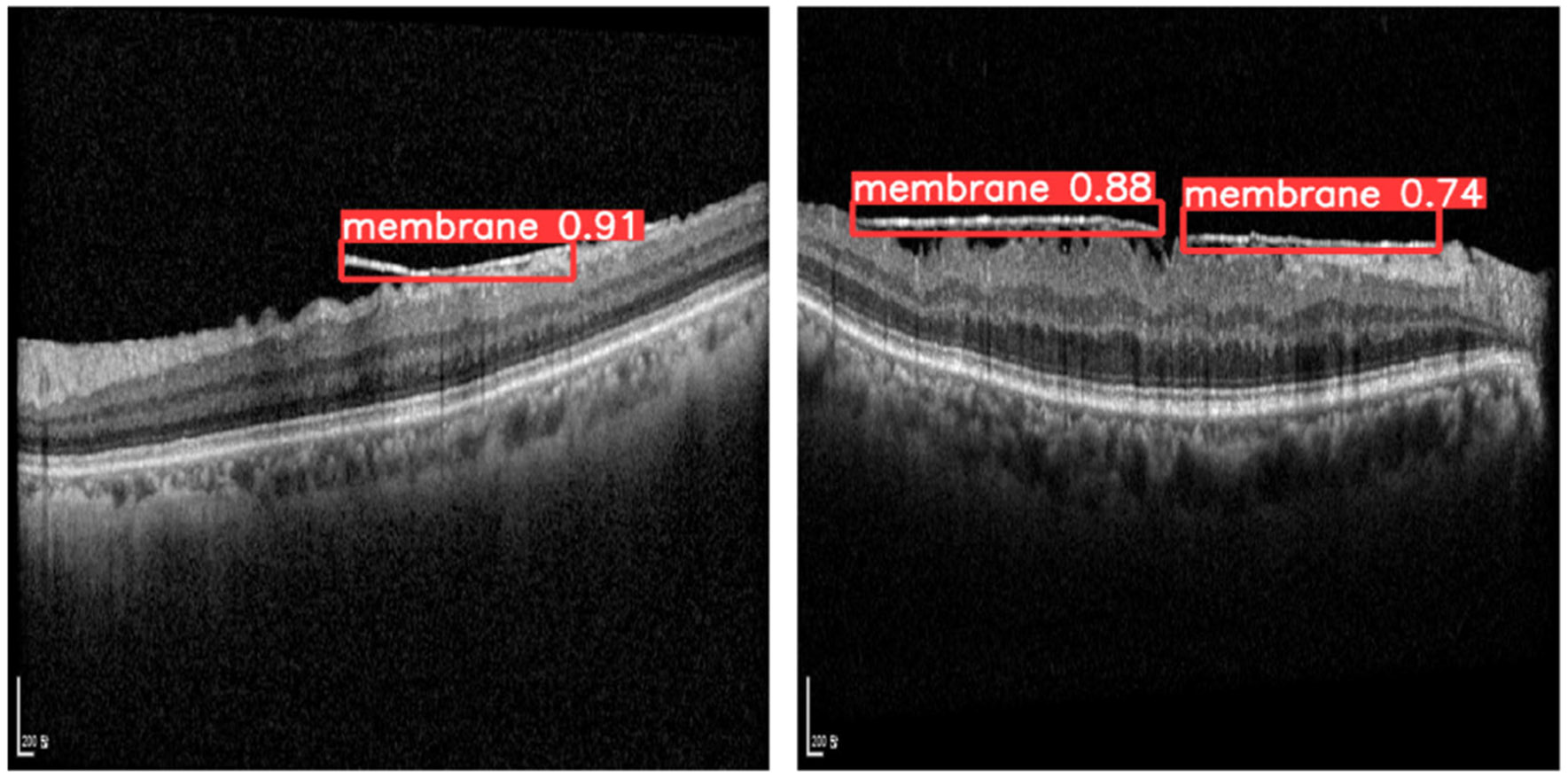
ERM detection results in B-scan images by YOLOv11x. The bounding boxes indicate the location of the ERM, along with the object ID and the probability of correctly predicting ERM.

Due to its superior detection performance and the quality of its resulting projection images, we employed YOLOv11x in the subsequent ERM projection and quantification experiments. FIGURE 4 presents two representative examples of ERM detection results on B-scan images using the trained YOLOv11x model.

## V. ERM PROJECTION FROM B-SCAN IMAGES

In this section, we explain how to generate an ERM projection image, referred to as EPI (ERM Projection Image) hereafter, from the ERM detection results obtained from B-scan images using YOLOv11x, which achieved the highest detection performance in our experiments.

The ERM detection results by YOLOv11x provide spatial location information of ERM across 50 B-scan images. These locations are then synthesized onto the SD-OCT en face image to create an “ERM Projection Image” that visually summarizes the entire ERM area on the retinal surface.

A B-scan image depicts the retinal layer structure at a specific cross-sectional location. The presence of ERM in a B-scan thus indicates ERM at the corresponding location in the en face view. Accordingly, the ERM detected in each B-scan is projected onto the en face image at its anatomically aligned position.

As illustrated in FIGURE 4, ERMs are identified using bounding boxes in the B-scan images. Among the 50 B-scans, only those with positive ERM detection are used to compose the en face image showing the comprehensive shape and extent of ERM.

**FIGURE 5.**
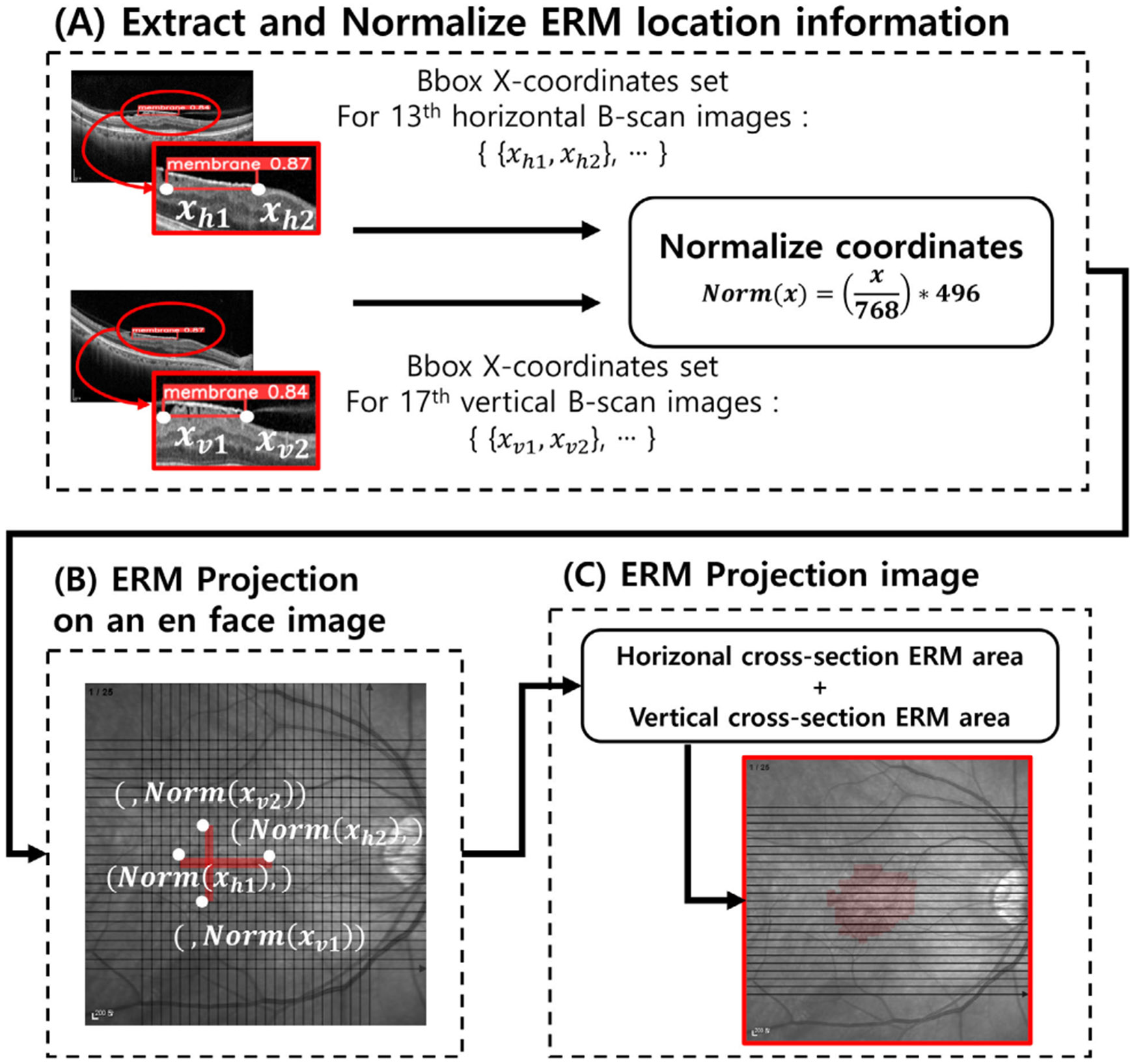
3 steps to create an EPI.

FIGURE 5 illustrates the three-step process of generating the EPI:

- Step (A) and (B) show examples of processing the 13th horizontal and 17th vertical B-scan images, respectively.
- Step (C) merges the ERM detection results from all 25 vertical and 25 horizontal B-scans into a final EPI.

Each step is described in detail below.

### A. EXTRACT AND NORMALIZE ERM LOCATION INFORMATION

First, we extract the x-coordinates of the bounding box endpoints from the YOLOv11x detection results. These indicate the lateral span of ERM in each B-scan. We then normalize these coordinates to map them onto the en face image, which differs in resolution.

In our dataset, the B-scan resolution is 768 × 496 pixels, while the en face image is 496 × 496 pixels. Therefore, normalization is required to align the spatial range of ERM between modalities.

The mapping is calculated by:

(1) $$x_{\text{enface}}=\left(\frac{x_{\text{Bscan}}}{W_{\text{Bscan}}}\right)\times W_{\text{enface}}$$

where $x_{\text{Bscan}}$ is the x-coordinate in the B-scan, $W_{\text{Bscan}}=768$, and $W_{\text{enface}}\;=\;496$. For vertical B-scans, the result is projected onto the y-axis of the en face image.

This normalization process is depicted in Step (A) of FIGURE 5, which converts raw B-scan coordinates into en face coordinates. Applying this to all 50 B-scans (25 horizontal, 25 vertical) yields a full set of normalized ERM ranges.

### B. ERM PROJECTION ONTO THE EN FACE IMAGE

In Step (B) of FIGURE 5, each ERM detection is projected onto the en face image according to its corresponding scan line. Horizontal B-scans use the normalized x-coordinates directly, while vertical B-scans map them to the y-axis.

This mapping aligns each ERM region with the correct anatomical orientation, as shown in the en face image where scan lines are indicated in black (see FIGURE 2).

To preserve underlying structural information such as vasculature, we use translucent red rectangles for the ERM projections. This enhances diagnostic visibility without obscuring important context.

### C. FINAL ERM PROJECTION IMAGE (EPI)

Step (C) of FIGURE 5 merges the projected ERM areas from all B-scans to generate a final en face image: the EPI.

By aggregating ERM data across 50 scans, the EPI visualizes the overall distribution and morphology of the membrane, offering a highly intuitive view for clinical interpretation.

Compared to conventional en face images from SD-OCT or SS-OCT, which often fail to depict ERM clearly, the EPI provides high-resolution, layer-accurate visualization grounded in cross-sectional evidence.

This approach also significantly reduces the workload for clinicians. Traditional ERM assessment requires browsing through dozens of B-scans, whereas the EPI offers a one-shot comprehensive view. Additionally, it enables visibility of ERM that might be invisible in SS-OCT en face images.

**FIGURE 6.**
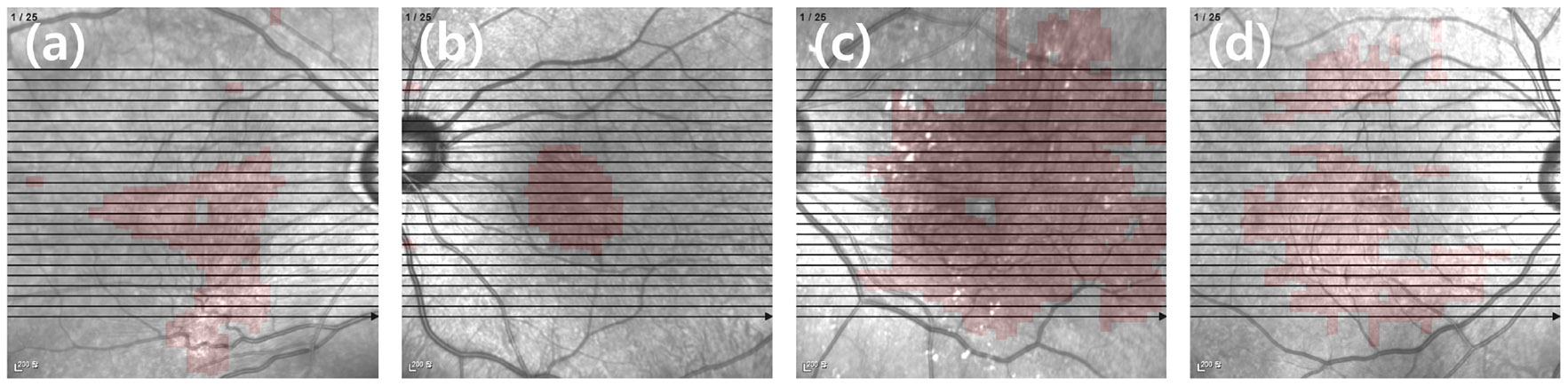
EPI: en face image with the entire area of the ERM displayed.

FIGURE 6 displays EPIs from four different patients. These examples illustrate the consistency and clarity of the proposed method.

In summary, the EPI:

- Provides an intuitive, high-fidelity visualization of ERM distribution,
- Outperforms SS-OCT en face images in clarity and reliability,
- Offers a fast, low-cost, and clinically meaningful tool for ERM assessment and preoperative planning,
- Aligns well with the surgeon’s intraoperative view, potentially reducing surgical time and complexity.

As such, the EPI has strong potential to shift the paradigm in ERM diagnosis and is suitable for integration into routine ophthalmic practice.

## VI. ERM QUANTIFICATION

Quantifying the ERM is essential for evaluating disease progression and determining appropriate treatment strategies.

En face images derived from SS-OCT often allow for identification and quantification of ERM. However, en face images from SD-OCT typically fail to depict the ERM area clearly, making accurate quantification challenging.

In this study, we utilize the ERM Projection Image (EPI), generated based on ERM detection results from B-scan images using YOLOv11x, to approximate the ERM area on SD-OCT en face images with high accuracy and at significantly lower cost than SS-OCT.

In a typical OCT setup, the imaging area and en face resolution are fixed, enabling conversion of pixel counts to physical area (*μm*^2^). The SD-OCT used in this study provides en face images of size 496 × 496, where every 10 pixels correspond to 200 *μm*, implying each pixel represents a 20 *μm* length.

Thus, each pixel covers an area of 400 *μm*^2^ (0.0004 *mm*^2^). The total ERM area is approximated by counting the number of pixels identified as ERM in the EPI.

TABLE 3 summarizes the quantified ERM areas from the four EPIs shown in FIGURE 6, including both absolute area and area ratio with respect to the entire en face image.

The ERM ratio indicates the proportion of the en face image area covered by ERM.

This quantification method shows that EPI enables effective ERM area estimation using SD-OCT, which has traditionally been limited in this regard.

Quantitative assessment of ERM contributes to evaluating disease severity, surgical planning by identifying target areas, and post-operative monitoring. This enables a more objective, data-driven approach to ERM treatment.

## VII. ASSOCIATION QUANTIFICATION OF ERM PROJECTION IMAGE BETWEEN THICKNESS MAP

As described in Section III, retinal thickness is critical information in the diagnosis of ERM because the area of retinal damage caused by ERM, which affects the treatment plan, is determined by the thickness of the retina. However, ERM is not the only factor that influences retinal thickness. Many other factors affect retinal thickness, leading to potential confusion between ERM and other eye diseases. For minimizing such confusion, multiple B-scan images and the retinal thickness map must be cross-referenced to accurately assess the association between ERM and retinal thickness The overlap area between the ERM and the retina that exceeds a specific thickness threshold serves as the basis for determining ERM treatment plans. Thus, in this paper, the association between ERM and retinal thickness is defined by the degree of overlap between the ERM and the retina that exceeds the thickness threshold. Cross-referencing B-scan images and the retinal thickness map on en face retinal images, as mentioned earlier, is time-consuming and inconvenient for ophthalmologists. The association between ERM and retinal thickness has not been quantified, making it difficult to standardize or rationalize the diagnostic criteria for ERM treatment.

In this section, we introduce an efficient and intuitive method for quantifying the association between retinal thickness and ERM on EPIs. The association quantification method that we present here can prevent ERM diagnosis confusion caused by retinal thickness maps. It can also provide quantifiable information for standardizing the ERM treatment plan based on the numerically quantified degree of association between ERM and retinal thickness, allowing the ophthalmologist to provide a rational basis for their treatment plans.

### A. ERM DIAGNOSIS BY CONTRASTING EPI AGAINST RETINAL THICKNESS MAP

This sub-section gives why to reason the association between ERM and the retinal thickness map. The EPI and retinal thickness map contain ERM area and retinal thickness information, respectively, in the same en face image. Such comparison enables the comparison of both datasets within the same view, allowing for an intuitive contrast between the ERM area and retinal thickness. This approach is significantly faster and more intuitive than diagnostics based on multiple B-scan images and retinal thickness maps.

**FIGURE 7.**
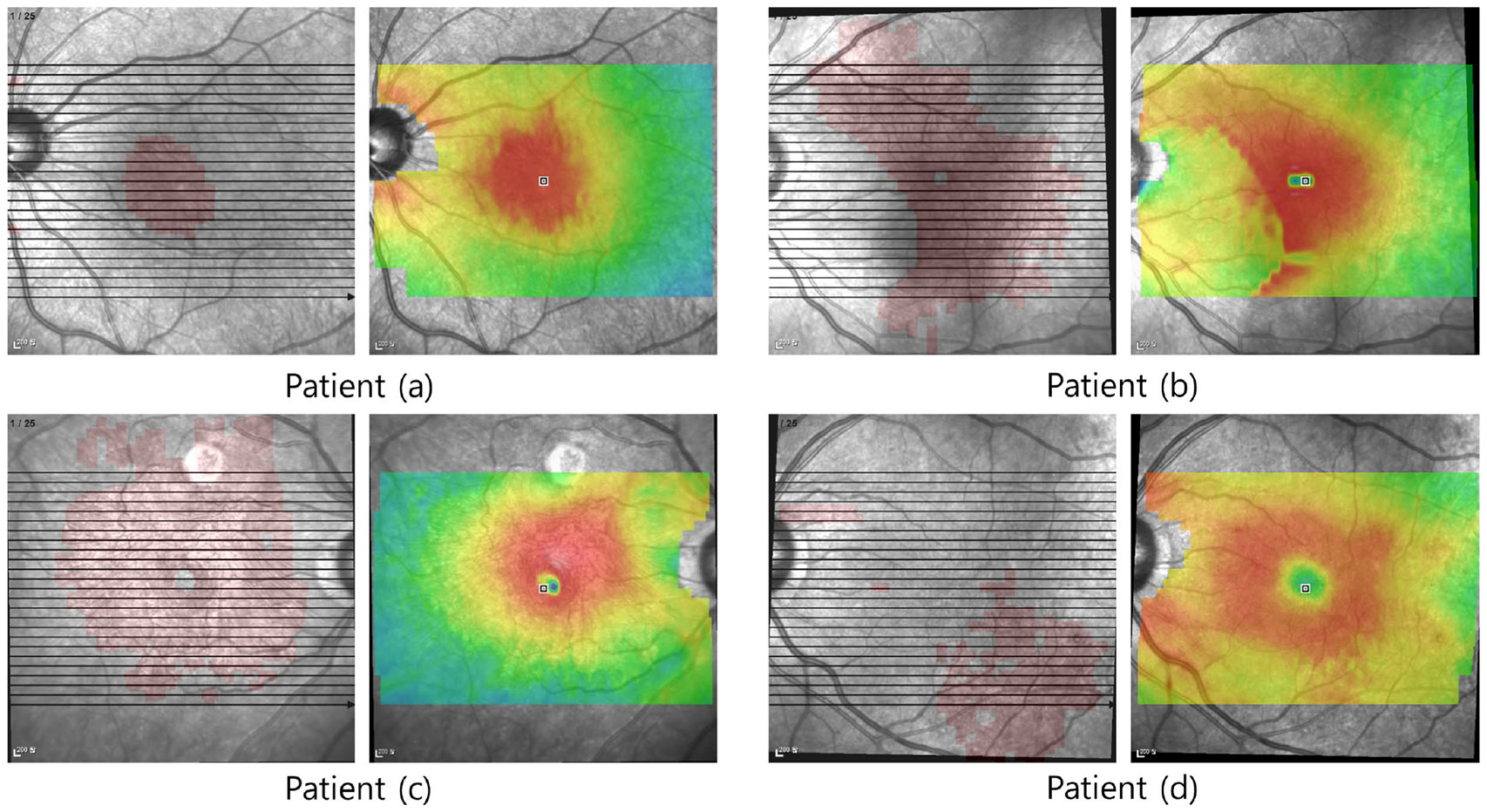
A set of EPIs and retinal thickness maps of four ophthalmology patients.

The EPI shows the ERM area of the retina, while the retinal thickness map shows the thickness distribution of the retina. Both are presented in the same view, an en face perspective of the retina, allowing for intuitive comparison between the ERM area and retinal thickness distribution and a more efficient planning of ERM treatment based on such comparison. In this sub-section, we visualize EPIs created by our method and the corresponding thickness map from SD-OCT, demonstrating how intuitive their comparison is. FIGURE 7 shows the EPIs and retinal thickness map from four patients. The retinal thickness maps in FIGURE 7 highlight retinal areas in deep red, indicating regions that may require medical treatment. Meanwhile, the green and yellow areas of the en face retinal image have less thickness compared to the red areas. Retinal areas that overlap with both the ERM identified by the EPIs and the deep red areas of the thickness map more likely indicate ERM-induced thickening of the retina.

The EPIs and en face retinal images of patients in FIGURE 7(a), (b), and (c) show that most ERM areas in the EPIs overlap with the thick retina in deep red areas. This likely indicates that the thickness of the retina has increased due to the ERM, and we would say that they have a strong association. On the other hand, the ERM in the EPI and the thick area of the retina in FIGURE 7(d) barely overlap with each other, meaning that the thickness of the retina is rarely affected by ERM, but by other factors. For example, retinal thickening can be caused by other eye diseases, or the retina may remain thick even after the eye has recovered from appropriate ERM treatment. In this case, we can say that the association between ERM and retinal thickening is not strong.

In addition to visually contrasting ERM and retinal thickness, we provide a quantification method to demonstrate the association between them by calculating the overlap between the EPI’s ERM area and the thickened retina. In this way, diagnosing ERM by contrasting its area with the thickened retina can be standardized and rationalized.

In the following sections, we will outline the process for quantifying this association and discuss the results obtained through this method.

### B. QUANTIFYING THE ASSOCIATION BETWEEN ERM AND RETINAL THICKNESS

The quantification process consists of three steps: First, we calculate the retinal area that needs to be considered for treatment based on the thickness map. Second, we compute the ratio of the overlap area relative to the sum of the ERM area and the total retinal area that requires consideration based on thickness. We refer to the overlap ratio as IoU (Intersection over Unit). Finally, we compute the association score based on the IoU.

#### 1) RETINA THICKNESS DISCRETIZATION

First, we identify the retinal area within the thickness map that needs to be considered for treatment. The retinal thickness map visually represents the thickness of the retinal layers through color coding within the en face image. This map displays the distribution of retinal thickness in contour form, using different colors to indicate varying thicknesses. FIGURE 3 shows the color distribution based on thickness, with thicknesses exceeding 500*μm* represented in white, while those below 500*μm* are depicted in a gradient starting from red and transitioning through yellow and green. Typically, ophthalmologists focus on retinal thickness values between 220*μm* and 500*μm* when examining the retinal thickness map. Particularly, retinal areas with a thickness of 400-500 *μm* are highlighted in red, serving as an important indicator of potential nerve fiber layer damage including ERM. In areas where ERM is diagnosed, the thicker the retinal layer, the more extensive the treatment required.

In this paper, we focus on the retinal areas within the 220*μm* to 500*μm* range as the first step in quantifying the association between ERM and retinal thickness. This range is further subdivided into nine evenly spaced classes, with each class representing a specific range of retinal thickness. We assign different weights to each retinal thickness region to reflect the potential severity of the damage corresponding to its thickness. This approach allows us to account for the seriousness of thickness changes and their clinical significance, facilitating more detailed analysis and accurate clinical decision-making.

**FIGURE 8.**
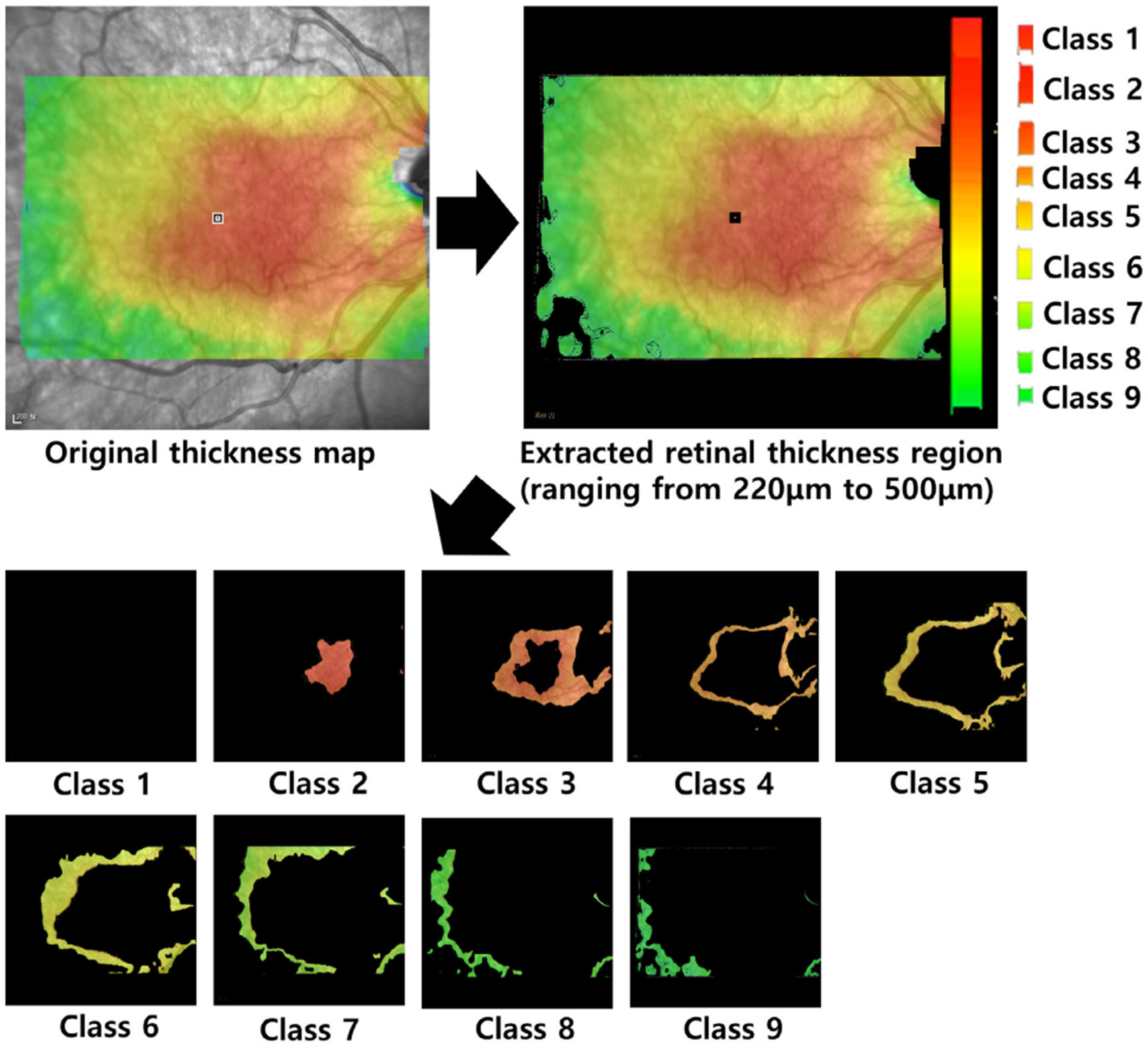
Retina Thickness Discretization into 9 Classes.

FIGURE 8 shows each steps of the aforementioned process: The first en face image shows the retinal area where thickness is measured and quantified. The second figure displays the retinal area with thicknesses between 220*μm* and 500*μm*, extracted from the first en face image. On the right of the second figure, the color distribution is shown according to the retinal thicknesses. At the bottom, individual retinal areas classified into 9 categories are shown, extracted from the second en face images.

#### 2) QUANTIFICATION OF ASSOCIATION BETWEEN THICKENED RETINA AND ERM FOR TREATMENT

The quantification of the association between thickened retina and ERM areas for treatment is done in two steps: 1) calculating the ratio of the overlap between the thickened retina and ERM for treatment, and 2) evaluating the association based on this ratio.

In order to the overlap ratio between the thickened retina and ERM, we define the Intersection Over Union (IoU) as follows:

*Definition 1:* : Intersection over Union(IoU)

$$IoU(A,B)=\frac{A\cap B}{A\cup B}$$

where A and B are areas that may be overlap with each other. For example, they are the thickened retina area or the ERM area of EPIs.

For the two areas, this ratio provides a value that helps assess how closely the identified ERM areas match to the retinal thickness area. The higher the IoU between the ERM area and the thickened retina area, the more the retinal thickness is influenced by the ERM.

The association quantification method we define in this paper calculates the degree of ERM’s impact on retinal thickness on a scale from 0 to 1. A higher value indicates a stronger impact of ERM on retinal thickness changes. It is designed to reflect the clinical significance of retinal thickness, giving greater weight to thicker areas, as they often signify more severe disease. This approach emphasizes the crucial role that thickened retina plays in determining the treatment plan for ERM.

As mentioned earlier, the thickened retina that ophthalmologists consider for treatment ranges from 220 *μm* to 500 *μm*, and we divide this range into 9 intervals. For a given ERM area and the corresponding retinal map, the association is computed by assigning different weights to each of these 9 thickness intervals.

The association between an ERM area and the retina thickness map is evaluated by an association score ($ASS_{score}$) computed as follows:

(2) $$ASS_{score}=\frac{1}{N}\sum_{i=1}^{N}\left(W_{i}\times IoU(A_{ERM},\sum_{j=1}^{i}A_{ret\_thick_{j}})\right)$$

where N is the number of retina thickness maps representing different thickness levels (1)-9), $A_{ERM}$ is the area of ERM in an EPI. $A_{ret_{t}hick_{j}}$ represents the area of a specific thickness level j in the retina thickness map. For example, $A_{ret_{t}hick_{2}}$ denotes the thickened retina area of Class 2 in FIGURE 8.

$W_{i}$ is defined in an arbitrary way in this paper because there is no previous study on the impact of ERM on the retina thickness. Here $W_{i}$ computed by the following:

(3) $$W_{i}=-0.01\cdot i^{2}-0.07\cdot i+1.666$$

such that $\sum_{i=1}^{9}W_{i}=1$ and that the thickened retinal areas in Levels 1-3 are more influenced by ERM than those in Levels 7-9.

The Association Score ($ASS_{score}$) gives greater weight to thicker retinal area when calculating the association with ERM, but as thinner retinal regions are progressively considered, the association with the thicker retinal areas is gradually reduced. It reflects the clinical significance of thicker retinal regions in indicating more severe symptoms of the disease, while still accounting for the overall retinal thickness distribution, including thinner regions.

This approach ensures a comprehensive evaluation of retinal thickness changes. Initially, the thicker regions are given more emphasis, but as the thinner retinal regions are added, the relative influence of the thicker regions decreases. This allows for a more balanced analysis that considers both the thicker and thinner areas of the retina.

Therefore, the Association Score reflects the clinical importance of each retinal thickness class while maintaining a balance between the influence of thicker and thinner retinal regions. This comprehensive approach enables a more accurate assessment of how ERM contributes to changes in retinal thickness.

TABLE 4 shows the computation results of the association score for EPIs and the retinal thickness maps in FIGURE 7. As the association score is close to 1, the impact of ERM on the retina’s thickening gets high. In FIGURE 7, patients (a), (b), and (c) show a high association, aligning with visual diagnosis outcomes. Conversely, patient (d) exhibits a lower correlation, indicating that factors other than ERM might be impairing retinal thickness, or the impairment is not severe. Therefore, specialists should consider the possibility of other diseases and perhaps conduct further tests or establish a more definitive diagnosis regarding eye damage due to ERM. In fact, the impairment in retinal thickness in patient (d) stems from diabetes, not ERM, emphasizing the weak association between ERM and retinal thickness.

## VIII. EVALUATION OF MODEL RELIABILITY UNDER CLINICAL INTERPRETATION

To validate the clinical reliability of the proposed ERM (Epiretinal Membrane) quantification model, two board-certified retina specialists—**Expert 1)** and **Expert 2**—independently assess the model outputs for *clinical accuracy* and *acceptability*. Unlike SS-OCT or thickness-based maps, which provide only indirect structural cues, the proposed evaluation directly reflects the physicians’ real-world interpretation of ERM morphology and extent. This expert-based assessment aims to determine whether the model’s detections are consistent with human clinical judgment, thereby establishing a practical benchmark for evaluating model reliability in actual diagnostic contexts.

### A. EVALUATION PROCEDURE AND DATASET

This clinical validation is designed to assess the proposed ERM (Epiretinal Membrane) quantification model under real-world diagnostic conditions.

Two board-certified retina specialists, **Expert 1** and **Expert 2**, independently evaluate a total of 30 ERM patient cases based on SD-OCT data and thickness map data. For each case, the experts rate the following statement on a 5-point Likert scale to assess both *clinical accuracy* and *clinical acceptability* of the model output:

“The model output is clinically accurate and requires no further correction.”

The Likert scale is defined as shown in TABLE 5. The assigned scores are numerically encoded and analyzed using correlation and agreement metrics to quantitatively evaluate both the model’s *clinical reliability* and the level of *expert acceptability*. This dual assessment framework allows the evaluation to capture not only how accurately the model reflects the true ERM morphology but also how readily its results can be accepted in real clinical workflows.

### B. ANALYSIS METRICS AND METHOD

To quantitatively evaluate both the *clinical reliability* of the proposed ERM quantification model and the level of *expert acceptability*, a set of complementary statistical metrics are employed. These metrics assess not only the inter-expert consistency in their evaluations but also the overall clinical acceptance of the model’s outputs. The definitions and interpretation criteria of these metrics are summarized in TABLE 6.

These indicators are widely adopted in medical image annotation and observer study analysis, allowing subjective expert evaluations to be quantified in terms of consistency, reproducibility, and acceptance. Specifically, the Pearson and Spearman correlation coefficients measure directional and rank-based consistency, the quadratic weighted Cohen’s *κ* and the intraclass correlation coefficient (ICC[2,1]) evaluate absolute reliability, and the Bland–Altman analysis examines systematic bias and limits of agreement (LoA) between experts. In addition, clinical acceptability indicators—such as the Average Acceptability Score (AAS), Acceptability Rate (AR), and Net Acceptance Score (NAS)—are used to quantify how strongly the experts agree that the model outputs are clinically reliable and usable without further correction.

This combined evaluation framework enables the quantitative validation of the proposed model from two complementary perspectives: (1) the reproducibility and agreement between human experts, and (2) the degree of clinical acceptance that the model’s EPI-based ERM quantification achieves in real diagnostic practice.

### C. RESULTS AND INTERPRETATION

As summarized in TABLE 7, the statistical agreement between the two retina specialists is consistently high across all metrics. The Pearson correlation coefficient (*r* = 0.939) and Spearman rank correlation (*ρ* = 0.909) indicate strong linear and ordinal consistency between the evaluators. The quadratic weighted Cohen’s *κ* = 0.886 and ICC(2,1) = 0.939 (95% CI: 0.90–0.97) both demonstrate *excellent reliability*. Bland–Altman analysis shows a mean difference of −0.033 ± 0.20, with limits of agreement (LoA) from [−0.410, +0.344], suggesting negligible systematic bias.

In terms of clinical acceptability, both ophthalmologists rated most cases as either *Strongly agree* or *Agree*, reflecting high confidence in the model’s EPI-based ERM quantification results. The average Likert scores were nearly identical between raters (Expert 1: 4.47 ± 0.57, Expert 2: 4.50 ± 0.51), yielding an overall mean of 4.48. The Acceptability Rate (AR; score ≥ 4) reached 85.0% (Expert 1: 83.3%, Expert 2: 86.7%), and the Net Acceptance Score (NAS) was +0.70, indicating that clinically acceptable outputs far outnumbered those requiring revision. Cohen’s *κ* for binary accept/reject decisions was 0.87, confirming excellent agreement on clinical acceptability judgments.

Overall, all reliability metrics (ICC, *κ, r, ρ*) approximate 0.9, and all acceptability indices (AAS, AR, NAS) indicate high clinical confidence in the model’s results. These findings collectively demonstrate that the proposed ERM quantification model produces results that are not only statistically consistent between experts but also *clinically acceptable and reliable for real-world diagnostic use*.

### D. LIMITATIONS AND CLINICAL IMPLICATIONS

Several limitations should be acknowledged in this study. First, the evaluation involves only two board-certified retina specialists (Expert 1 and Expert 2), which may not fully represent the variability of clinical interpretations across a broader population of ophthalmologists. Second, the 5-point Likert scale employed in this study provides ordinal rather than continuous data, introducing statistical constraints when conducting parametric or regression-based analyses.

Third, even in real clinical settings, delineating the exact boundary of an epiretinal membrane (ERM) using SS-OCT, thickness maps, or SD-OCT B-scan images alone remains inherently difficult. This challenge arises from substantial inter-patient variability in membrane reflectivity, transparency, and adhesion characteristics to the retinal surface. Consequently, instead of establishing an absolute anatomical or segmentation-based *ground truth*, this study focuses on evaluating model reliability and clinical validity through the degree of consensus and acceptability among retina specialists.

Furthermore, while the evaluation of 30 representative ERM cases provides a valuable preliminary basis for validating the model’s feasibility and reliability, the sample size remains limited. Future studies involving a larger and more diverse patient cohort, ideally across multiple centers, would be beneficial to further substantiate and generalize the present findings.

Nevertheless, the proposed ERM quantification model demonstrates relatively high clinical accuracy and strong expert acceptability. The inter-expert agreement remains excellent (ICC = 0.94, *κ* = 0.89), suggesting that the method attains a reproducible level of reliability under clinical conditions. Moreover, the findings indicate that the model may capture the pathological morphology of ERM more precisely than conventional OCT-based thickness or projection indices, highlighting its potential as a practical and interpretable tool for future clinical decision support applications.

## IX. DISCUSSION

In this section, we discuss three issues that we don’t resolve in this paper.

### A. GAP BETWEEN CROSS SECTIONS

SD-OCT captures B-scan images by shifting at regular intervals both vertically and horizontally across the retina. For this reason, the accuracy of the ERM quantification we propose for a given vertical and horizontal retina area depends on the resolution of the cross-sectional scans. That is, the smaller the gap between each cross-sectional scan, the more accurate the ERM quantification.

### B. ERM SEGMENTATION

The length measurement of the ERM on vertical or horizontal cross-section images is based on the size of the bounding box for the target object in YOLO. However, due to highly curved retinal monolayers or significantly curved ERMs, the ERM length derived from YOLO’s bounding box may be shorter than the actual ERM length, potentially leading to errors in the patient treatment plan. Since the YOLO framework defines rectangular regions around curved or irregular membranes, the estimated ERM area and length may underestimate the true morphology, particularly in cases with folded or multilayered membranes. This geometric simplification can introduce minor quantification bias in both area- and thickness-based analyses.

To address this limitation, future integration with segmentation-based refinement is planned to enhance morphological fidelity. In particular, region-based approaches such as Mask R-CNN or U-Net can delineate pixel-level boundaries and better capture the complex curvature of ERMs, providing more anatomically accurate quantification.

This issue can be mitigated by the method proposed by Lo et al. (2020) [21], which enables a more accurate estimation of an object’s shape by precisely delineating its area within an image. It is a segmentation approach that allows for exact measurements of curved lengths, accommodating the irregularities of bent ERMs and curved retinal layers. Incorporating such segmentation refinement within the proposed EPI framework is expected to reduce area underestimation and improve quantitative consistency across patients. Although segmentation requires more robust data and annotations, it enhances measurement accuracy from the images, thereby improving the quality of EPIs and the overall clinical reliability of ERM assessment.

### C. PRE-CLINICAL VALIDATION

In clinical practice, all medical software must undergo rigorous validation to ensure its performance and accuracy before deployment. The same applies to our ERM quantification algorithm. Ideally, validation of ERM area measurements should be performed using en face images obtained from swept-source optical coherence tomography (SS-OCT), which provide the most accurate delineation of ERMs currently available. However, in our research setting, acquiring sufficient SS-OCT datasets corresponding to our spectral domain OCT (SD-OCT) data used for model training and testing was not feasible due to limited resources and data-sharing constraints. This limitation made it difficult to directly compare our method with advanced techniques such as SS-OCT-based quantification. To address this, we leveraged the proven accuracy of the YOLO algorithm in detecting ERMs in B-scan images as an indirect indicator of reliability in generating ERM projection images. Additionally, to supplement this indirect validation, we consulted a board-certified ophthalmologist who confirmed that our algorithm not only produced high-accuracy delineation of ERM regions but also offered superior efficiency in terms of both cost and time compared to SS-OCT. We acknowledge, however, that this approach does not replace the value of direct comparison with SS-OCT or other state-of-the-art methods. A comprehensive, objective comparison remains an important next step. Therefore, as part of our future work, we are considering further validation using SS-OCT data to more robustly evaluate and benchmark our method’s clinical utility.

## X. CONCLUSION

Epiretinal membrane (ERM) is a common ocular condition that requires precise and efficient diagnostic assessment. While spectral-domain optical coherence tomography (SD-OCT) is widely used in clinical practice, it provides only sectional information, making it difficult to evaluate the overall extent of ERM. In contrast, swept-source OCT (SS-OCT) enables en face visualization of ERM morphology but remains limited in clinical use due to its cost, complexity, and data-processing burden. To address these limitations, this study proposed a deep learning–based ERM quantification framework that reconstructs en face *Epiretinal Projection Images (EPI)* from SD-OCT B-scans, effectively reproducing the advantages of SS-OCT using more accessible SD-OCT data.

The proposed pipeline applies a YOLO-based detection model to identify ERM regions across multiple B-scan slices and combines them into a single en face projection map. This process enables intuitive visualization and quantitative assessment of ERM while significantly reducing the time and manual effort required for diagnosis. Furthermore, the method quantitatively associates EPI-derived ERM areas with retinal thickness profiles, allowing for more objective and interpretable evaluation of ERM severity and spatial influence.

To verify clinical reliability and expert acceptability, two board-certified retina specialists independently evaluated 30 ERM cases. Their assessments showed excellent inter-expert agreement (ICC ≈ 0.94, *κ* ≈ 0.89) and high clinical acceptability (AAS = 4.48, AR = 85.0%), confirming that the proposed EPI-based quantification closely aligns with expert clinical judgment.

In summary, the proposed framework provides a cost-effective and clinically interpretable approach for ERM detection and quantification using standard SD-OCT systems. It bridges the diagnostic gap between SD-OCT and SS-OCT, offering a scalable solution that enhances both diagnostic efficiency and objectivity. Future studies will extend validation across larger and more diverse clinical datasets to further establish its robustness and generalizability in real-world ophthalmic practice.

## Figures and Tables

**TABLE 1.** Performance evaluation of SD-OCT, SS-OCT, and SD-OCT with our techniques based on various criteria, including ease of observations, area quantification, diagnostic clarity, and cost.

Evaluation Factors	SD-OCT	SS-OCT	SD-OCT + Our Techniques
En-face ERM image	X	O	O
ERM Quantification	X	O	O
Thickness Evaluation	X	X	O
Cost	Low	High	Low
Data Size	Small	Large	Small
Processing Time	Short	Long	Short

**TABLE 2.** Performance comparison of YOLOv5, YOLOv8, and YOLOv11 models on ERM detection using SD-OCT B-scan images.

Model	Size	Params (M)	Train Dataset Size	Precision	Recall	mAP@50	mAP@50:95
YOLOv5	S	7.02	2200	0.752	0.703	0.722	0.423
			1300	0.694	0.642	0.664	0.376
	M	20.87	2200	0.783	0.734	0.752	0.444
			1300	0.723	0.685	0.701	0.396
	L	46.14	2200	0.813	0.762	0.784	0.463
			1300	0.745	0.704	0.726	0.414
	X	86.22	2200	0.836	0.784	0.802	0.485
			1300	0.763	0.725	0.743	0.437
YOLOv8	S	11.14	2200	0.781	0.736	0.764	0.447
			1300	0.723	0.676	0.701	0.393
	M	25.86	2200	0.813	0.762	0.791	0.466
			1300	0.748	0.705	0.724	0.412
	L	43.63	2200	0.844	0.792	0.823	0.482
			1300	0.774	0.731	0.754	0.436
	X	68.15	2200	0.867	0.814	0.842	0.504
			1300	0.793	0.752	0.772	0.454
YOLOv11	S	9.43	2200	0.804	0.752	0.783	0.468
			1300	0.746	0.692	0.714	0.417
	M	20.05	2200	0.846	0.794	0.821	0.493
			1300	0.774	0.736	0.757	0.443
	L	25.31	2200	0.873	0.823	0.854	0.524
			1300	0.807	0.773	0.793	0.476
	X	56.87	2200	0.902	0.857	0.882	0.556
			1300	0.836	0.803	0.826	0.507

**TABLE 3.** Quantification of ERM in four EPIs shown in FIGURE 6.

EPI	ERM area [*μm*^2^]	ERM ratio
Patient (a)	14,140,800	14.36%
Patient (b)	5,990,800	6.08%
Patient (c)	46,722,000	47.47%
Patient (d)	23,815,600	24.20%

**TABLE 4.** Quantify the correlations for the 4 patients in FIGURE 8.

Patients	(a)	(b)	(c)	(d)
Association Score	0.3531	0.3372	0.3477	0.1145

**TABLE 5.** Definition of the 5-point Likert Scale Used for Clinical Accuracy and Acceptability Evaluation.

Score	Description
5	*Strongly agree* — highly accurate and fully acceptable
4	*Agree* — minor deviations but clinically acceptable
3	*Moderate / Neutral* — partially accurate, requires review
2	*Disagree* — inaccurate, substantial revision needed
1	*Strongly disagree* — clinically unreliable or unusable

**TABLE 6.** Statistical and acceptability metrics used for expert agreement and model reliability evaluation.

Metric	Description	Interpretation Criteria
Pearson correlation (*r*)	Linear agreement between scores	*r* > 0.8: strong agreement
Spearman correlation (*ρ*)	Rank-based ordinal agreement	*ρ* > 0.8: high agreement
Quadratic weighted *κ*	Distance-weighted ordinal agreement	*κ* > 0.8: excellent agreement
ICC(2,1)	Absolute inter-rater reliability (two-way random)	ICC > 0.9: excellent reliability
Bland–Altman analysis	Mean bias and limits of agreement	Mean ≈ 0: negligible bias
AAS (Average Acceptability Score)	Mean of 5-point Likert ratings	Higher = better acceptability
AR (Acceptability Rate ≥4)	Proportion of “Agree” or “Strongly agree” responses	>80%: high clinical acceptance
NAS (Net Acceptance Score)	Balance of acceptance vs. rejection (#A−#R)∕N	Positive = acceptance dominant

**TABLE 7.** Summary of inter-expert agreement and clinical acceptability metrics.

Metric	Value	Interpretation
Pearson *r*	0.939	Very high linear agreement
Spearman *ρ*	0.909	Strong ordinal agreement
Weighted *κ*	0.886	Excellent agreement
ICC(2,1)	0.939 (95% CI: 0.90–0.97)	Excellent reliability
Mean difference (Bland–Altman)	−0.033 ± 0.20	Negligible systematic bias
Limits of agreement (LoA)	[−0.410, +0.344]	Clinically acceptable range
AAS (Mean Likert Score)	4.48 (Expert 1: 4.47, Expert 2: 4.50)	High clinical acceptability
AR (Accept Rate ≥4)	85.0% (E1: 83.3%, E2: 86.7%)	Majority of outputs clinically accepted
NAS	+0.70	Acceptance strongly outweighs rejection
Cohen’s *κ* (Accept/Reject)	0.87	Excellent agreement on acceptability
